# Effects of Tuina combined with traditional Chinese exercises on function disability in patients with lumbar disc herniation: a multicentre, randomised, controlled clinical trial

**DOI:** 10.3389/fneur.2025.1599301

**Published:** 2025-10-08

**Authors:** Yitao Liao, Yi Wang, Zhihong Fan, Qingguang Zhu, Xin Zhou, Dandan He, Chao Li, Xian Zhang

**Affiliations:** ^1^Department of Graduate School, Nanjing University of Chinese Medicine, Nanjing, China; ^2^Department of Spine, Wuxi Affiliated Hospital of Nanjing University of Chinese Medicine, Wuxi, China; ^3^Yueyang Hospital of Integrative Medicine Affiliated with the Shanghai University of Traditional Chinese Medicine, Shangha, China; ^4^Jiangsu Provincial Research Institute of Chinese Medicine Schools, Nanjing, China

**Keywords:** lumbar disc herniation, Tuina therapy, traditional Chinese exercises, randomised controlled trial, function disability

## Abstract

**Background:**

Low back pain and leg pain are common symptoms of lumbar disc herniation (LDH), which predispose patients to walking dysfunction and affect their quality of life. Tuina and Traditional Chinese Exercises (TCEs) are often used in China as passive or active treatments to alleviate the symptoms of LDH in patients and to address disability. However, high-quality multicentre clinical trials evaluating the short- and long-term efficacy of Tuina combined with TCEs in the treatment of LDH are lacking.

**Methods:**

In a multicentre, randomised, controlled clinical trial, 166 patients with LDH were recruited from four centres and randomly assigned into two groups that were treated with TCEs and Tuina combined with TCEs. Each group received intervention 3 times in 1 week for 4 weeks, and efficacy was assessed at baseline, 4 weeks of treatment, 12 weeks of follow-up and 24 weeks of follow-up. The primary outcome indicator assessed was the Oswestry Disability Index (ODI), and the secondary outcome indicators were the Visual Analogue Scale (VAS), the Short Form of Quality of Life (SF-36) Scale, the Short-Form McGill Pain Questionnaire (SF-MPQ) Scale and gait analysis.

**Results:**

A total of 157 subjects completed the trial, and 9 were dislodged. After 4 weeks of intervention, the ODI mean value in the Tuina combined with TCE group was 16.31 (4.18), a decrease of 7.75 (95%, 6.88–8.62) from baseline. The mean value in the TCE group was 20.23 (3.43), a decrease of 3.79 (95%, 2.92–4.67) from baseline. The ODI scores were significantly lower in the Tuina combined with TCE group compared with the TCE group at weeks 4, 12 and 24, with mean differences of 3.92 (95%, 2.75–5.09, *p* < 0.001), 2.90 (95%, 1.63–4.18, *p* < 0.001) and 3.03 (95%, 1.70–4.36, *p* < 0.001), respectively. The Tuina combined with TCE group also performed significantly better than the TCE group in the VAS, SF-MPQ, SF-36 and gait analysis.

**Conclusion:**

Tuina combined with TCE therapy can effectively improve function disability, pain, quality of life and pace of step in patients with LDH, and the combined therapy is superior to single TCE therapy.

**Clinical trial registration:**

ChiCTR2300077361; https://www.chictr.org.cn/showproj.html?proj=209956.

## 1 Introduction

Lumbar Disc Herniation (LDH) commonly manifests as low back pain (LBP), sciatica, muscle weakness, and in severe cases, neurological deficits such as orchialgia, ultimately leading to functional impairment and disability (1, 2). LDH mainly occurs in young and middle-aged people, but with the change of lifestyle and work style, the incidence of LDH tends to increase, and the age of onset tends to be younger (3). The prevalence rates of lumbar disc degeneration and LDH in children and adolescents were 2.2 and 5.8%, respectively, in an image-based epidemiologic survey (4). LDH is strongly associated with severe disability, severely affects patients’ ability to work normally and raises their social costs; thus, choosing simpler, more effective treatment options is particularly important (5, 6).

Currently, the treatment of LDH is mainly categorised into conservative treatment and surgical treatment; most patients select conservative treatment, and their symptoms can be improved (7). Conservative treatments are subdivided into pharmacologic and nonpharmacologic therapies. Nonsteroidal anti-inflammatory drugs (NSAIDs) are frequently utilized to alleviate pain and inflammation associated with LDH, serving as analgesics for acute episodes (8). While NSAIDs can provide short-term relief for acute radicular pain in LDH, their efficacy for chronic LDH symptoms is limited, and prolonged use increases the risk of gastrointestinal complications (9, 10). Consequently, recent studies have shifted emphasis from pharmacologic and surgical options as first-line treatments, favoring nonpharmacologic therapies such as massage, rehabilitation, acupuncture, and chiropractic for LDH management (11–14).

Tuina is one of the Chinese specialty therapies, often widely used as a nonpharmacological analgesic therapy for a variety of diseases, and the current research frontiers are mainly in the relief of LBP (15). Tuina is effective in reducing pain for musculoskeletal conditions, improved circulation and lymphatic drainage, and induce immune system support (16–19). A study showed that nudging to relieve LBP may have an analgesic effect by modulating the dysfunctional areas of the brain that play an important role in regulating pain (20). Although massage can reduce pain, relieve local muscle spasms, improve body functions and regulate spinal balance, it can neither improve the stability of the lumbar spine and the muscle strength of the paravertebral muscles nor reduce the recurrence of pain (21). Traditional Chinese Exercises (TCEs) are uniquely Chinese workouts that nourish the body and emphasise the combination of movement, breathing and intention.

TCEs improve functional disability, balance and fall prevention, quality of life, stress anxiety, and cardiovascular health (22–25). However, high-quality, multicentre, randomised controlled trials evaluating the short- and long-term efficacy of Tuina combined with TCEs in the treatment of LDH are lacking; similarly, clinical trials demonstrating whether the combination is superior to treatment with TCEs alone are rare.

Therefore, in this paper, a multicentre, randomised, controlled clinical trial study was conducted through the treatment modality of Tuina combined with TCEs to evaluate the effects of Tuina combined with TCEs on functional disability, pain, quality of life and gait in patients with LDH, and to compare the difference in efficacy between the combined treatment and TCEs alone.

## 2 Methods

### 2.1 Study design

A multicentre randomised, controlled clinical trial study was conducted in four clinical centres. The Wuxi Hospital of Traditional Chinese Medicine was the main centre, and the Yueyang Hospital of Integrative Medicine affiliated with the Shanghai University of Traditional Chinese Medicine, the Wuxi Rehabilitation Hospital and the Wuxi Xinwu District Hospital of Traditional Chinese Medicine were the three subcentres. Patients were recruited through advertisements at each centre. Patients who met the inclusion criteria were randomly assigned to the TCE group and the Tuina combined with TCE group for a four-week intervention. Follow-up visits were conducted at the 12th and 24th weeks after the end of the intervention to complete data collection. The study was approved by the Ethics Committee of the Wuxi Hospital of Traditional Chinese Medicine (STHZG2023021301).

### 2.2 Participants

A total of 166 subjects were recruited for this study: 76 were from the Wuxi Hospital of Traditional Chinese Medicine, and 90 were enrolled in 30 subjects from each of the three subcentres. Baseline data such as age, gender, height and weight were obtained through interviews at the time of signing the informed consent form. The inclusion criteria were as follows: (1) age ≥18 years and ≤65 years with no gender restriction; (2) meeting the diagnostic criteria for LDH; (3) duration of the disease ≥3 months; (4) Visual Analogue Scale (VAS) scores >3 and ≤7; (5) voluntarily participation in the study and signed informed consent form. The exclusion criteria were as follows: (1) history of previous severe spinal trauma; (2) spinal bone tumours, tuberculosis and osteoporosis, as seen on imaging; (3) severe neurological deficits, such as cauda equina injury; (4) combined cardiovascular, cerebrovascular, hematopoietic, gastrointestinal, and other serious illnesses or psychiatric disorders; (5) other autoimmune diseases, anaphylactic disorders, and acute and chronic infections; (6) participation in other clinical trials within the last 3 months. Those with one of the above conditions cannot be included in this trial.

### 2.3 Randomisation, allocation concealment and blinding

Eligible participants were randomly assigned in a 1:1 ratio to receive either Tuina combined with TCE treatment (n = 83; 38 from the main centre and 45 from the three subcentres) or TCE treatment alone (n = 83; 38 from the main centre and 45 from the three subcentres). Block randomisation was employed by a statistical expert using SAS software (version 9.4, M_3; SAS Institute Inc., USA), randomization was stratified by enrollment site with a block size of 4. The random number table was securely maintained in Microsoft Excel by an independent researcher who was not involved in any other aspect of the study. Random numbers were placed in sequentially numbered, opaque, sealed envelopes to ensure allocation concealment. For each participant enrolled, the responsible researcher at each centre contacted the independent allocator, who revealed the group assignment by opening the corresponding envelope. This procedure ensured strict allocation concealment throughout the enrolment process.

### 2.4 Intervention

TCE and Tuina interventions were delivered by two designated licensed therapists at each participating centre, with each therapist responsible for only one specific intervention modality—either TCE or Tuina. To ensure consistency and minimize practitioner-related bias, all therapists underwent centralized training and certification organized by the main research centre prior to the start of the study. Only those who successfully completed this standardized training and passed a formal qualification assessment were permitted to provide treatment. Mandatory therapist qualifications included a valid professional license and a minimum of 5 years of documented clinical experience in their respective therapy (TCE or Tuina). A unified treatment protocol was established and implemented across all centres, and regular oversight from the main centre ensured protocol adherence. Prior to treatment initiation, all participants received a comprehensive explanation and live demonstration of the TCE protocol. To facilitate home practice and improve adherence, participants were provided with an instruction manual and standardized instructional videos specifying the form, duration, and frequency of each TCE movement. All interventions were administered three times per week for four consecutive weeks. Follow-up assessments were conducted at weeks 12 and 24. Throughout the study period, participants were instructed to maintain their normal daily routines and avoid any additional structured exercise outside of the assigned interventions.

### 2.5 TCE group

TCEs have a wide variety, and to select suitable TCE movements to be performed by patients with LDH, the Delphi method was used to screen four movements by means of an expert questionnaire in the prestudy period. The TCEs were selected from Baduanjin, Taiji and Wuqinxi, and minor modifications were made to accommodate the patients’ varying learning and exercise abilities. The program primarily encompassed the fourth movement of Baduanjin, the Yunshou of Taiji, Huju and Luben from Wuqinxi. Each exercise should last no less than 20 min. TCEs were instructed and taught thrice per week. Except for the first week when face-to-face instruction was mandatory, subjects could opt to receive instruction in person, by phone or by videoconference for the three following weeks, but one face-to-face meeting per week was required to confirm completion and progress.

### 2.6 Tuina combined with TCE group

The Tuina procedure was divided into localised muscle release and lumbar joint adjustment, and the whole process lasted approximately 20–30 min. Firstly, gentle rubbing and kneading were performed on both sides of the patient’s spine and buttocks using the fingertip surface, and then the local pain points or acupoints were pointed and pressed (e.g., BL23, BL24, BL25, BL40, and GB30). Following the completion of local muscle release, joint adjustments were done by utilising a lumbar blique-pulling manipulation and posterior lumbar extension. The lumbar blique-pulling manipulation was comparable to chiropractic manipulation, and the audible ‘click’ indicated the successful completion of the procedure. Finally, lumbar back extension was performed. The patient was initially placed in the prone position, and the therapist pressed one hand on the patient’s spinal pain and slightly lifted the patient’s lower limbs with the other hand. The therapist then waited for the patient’s lumbar muscles to relax. Next, the therapist quickly lifted the patient’s lower limbs up to 30°–40° and then placed them down immediately. Following Tuina, the patient should be permitted to rest in bed for approximately 10–15 min. The treatment was administered thrice per week. The training movements and methods employed by the TCEs were identical to those utilised in the control group.

### 2.7 Outcomes

The primary outcome indicator was the Oswestry Disability Index (ODI) scale (26), which was used to rate the patient’s dysfunction subjectively. The secondary outcome indicators were the Short Form of Quality of Life Scale (SF-36), the Short-Form McGill Pain Questionnaire Scale (SF-MPQ) and gait analysis parameters. The SF-36 consists of eight physical and mental dimensions to evaluate the quality of life of patients (27), and only two dimensions, namely physical function (PF) and mental health (MF), were used in this study. The SF-MPQ consists of three parts, namely pain rating index (PRI), VAS and present pain intensity (PPI) (28), which were employed to evaluate the patients’ pain status. Gait analysis was used to assess the walking function of the patients in terms of step length, cadence, stride length and double-stance phase (29), but it was only used to compare the efficacy of the patients before and after 4 weeks of the intervention.

All relevant items were confirmed, and the patients provided informed consent. The data were analysed by an independent statistician.

### 2.8 Statistical analysis

The sample size calculation was based on our prestudy. The mean and standard deviation (SD) of ODI after 4 weeks of intervention were 19.73 and 1.94 in the TCE group, respectively. The mean and SD of ODI after 4 weeks of intervention in the group of Tuina combined with TCEs were 15.63 and 2.59, respectively. α was taken as 0.05, and β was taken as 0.2, which was calculated to provide the sample size of 74 in each group. Considering the 20% dropout rate, the final sample size of each group sample size was 83, and the total sample size was 166.

All data were statistically analysed using IBM SPSS Statistics V.27.0. Descriptive statistics and histogram checks were used to determine whether the data were normally distributed, and the chi-square test was employed to compare the differences in baseline information between the two groups. For other continuous variables (age, weight, height and body mass index), comparisons between treatment groups were assessed using the independent sample t-test. The level of statistical significance was set at 5% (*p* < 0.05). The outcomes, including ODI, VAS, SF-MPQ (PRI), SF-36 (PF), and SF-36 (MH), were analyzed using repeated measures ANOVA. A Bonferroni correction was used to account for multiple comparisons. All analyses were conducted on an intention-to-treat (ITT) basis, with participants who did not complete the study assumed to have no change from baseline at all assessment points.

## 3 Results

### 3.1 Clinical assessment

**Figure 1:**
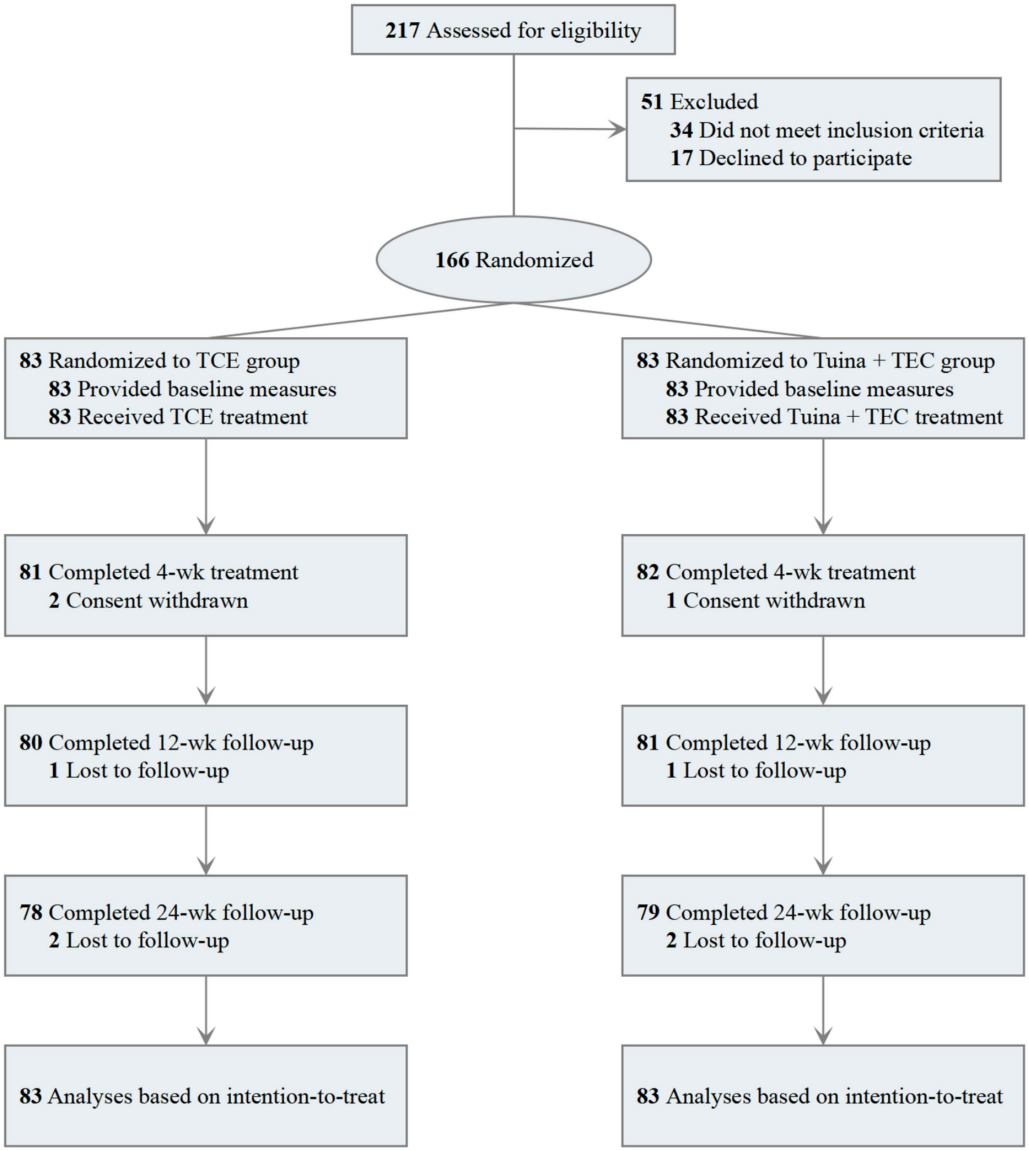
Flow chart of the study design.

Between March 1, 2023 and November 30, 2023, 217 participants were recruited at our four subcentres: 34 patients met the diagnosis of LDH but did not meet the inclusion criteria, 17 patients refused to sign the informed consent, and 166 patients met the inclusion criteria and agreed to sign the informed consent. These 166 subjects were randomly assigned to receive Tuina combined with TCEs (n = 78) or TCEs alone (n = 78). During the trial, 5 participants withdrew informed consent due to personal scheduling conflicts (1 from the Tuina + TCE group and 2 from the TCE group), and 6 participants were lost to follow-up (3 from each group), resulting in an overall dropout rate of approximately 5.4%. The dropout rate was 4.8% (4/83) in the Tuina + TCE group and 6.0% (5/83) in the TCE group. The distribution of dropouts was comparable between the two groups and did not significantly affect group balance, *p* > 0.05 (Figure 1). Baseline characteristics such as age, gender, height and weight were compared between subjects in the Tuina combined with TCE group and the TCE group, and the baseline characteristics of subjects in both groups were essentially similar (Table 1).

**Table 1:** Comparison of baseline characteristics between the two groups of subjects.

Characteristic	TCE group (*n* = 83)	Tuian+TCE group (n = 83)	*p* value
Age (years)	44.94 (12.16)	47.53 (10.83)	0.160
Gender (female/male)	41/42	43/40	0.758
Height (cm)	167.60 (8.31)	167.28 (8.54)	0.810
Weight (kg)	68.53 (12.66)	68.96 (15.06)	0.847
BMI (kg/cm^2^)	24.23 (2.81)	24.44 (3.69)	0.686
Duration of symptoms (month)	13.05 (24.36)	19.82 (39.66)	0.199
Level of the herniation (%)			
L4/L5	30 (36.1)	37 (44.6)	0.327
L5/S1	38 (45.8)	29 (34.9)	
L4/L5 and L5/S1	15 (18.1)	17 (20.5)	

Data are presented as mean (standard deviation). BMI, Body mass index.

**Figure 2:**
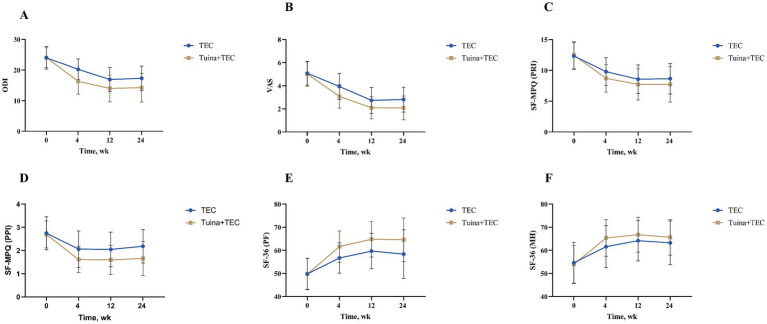
Changes in outcomes among groups over time. **(A)** ODI scores across time points for both TEC and Tuina+TEC groups **(B)** VAS scores over time for both groups **(C)** SF-MPQ (PRI) scores in both groups at various time points **(D)** SF-36 (PPI) scores across time for both groups **(E)** SF-36 (PF) scores measured over time for TEC and Tuina+TEC groups **(F)** SF-36 (MH) scores showing changes over time. Error bars are included for variability. ODI, Oswestry Disability Index; VAS, Visual Analogue Scale; SF-36: Short Form of Quality of Life; SF-MPQ: Short-Form McGill Pain Questionnaire; PRI, Pain rating index; PPI: Present pain intensity; PF, Physical function; MH, Mental health.

The main outcome indicators were statistically analysed. After 4 weeks of intervention, the ODI scores were lower than at baseline in the Tuina + TCE group and the TCE group. The mean value in the Tuina + TCE group was 16.31 (4.18), with a mean difference of 7.75 (95%, 6.88–8.62) from baseline. The mean value in the TCE group was 20.23 (3.43), with a mean difference of 3.79 (95%, 2.92–4.67) from baseline. The ODI scores were significantly lower in the Tuina + TCE group after 4 weeks of intervention compared with the TCE group, with a mean difference of 3.92 (95%, 2.75–5.09, *p* < 0.001). At 12-week and 24-week follow-ups, the Tuina + TCE group continued to have lower scores than the TCE group, with mean differences of 2.90 (95%, 1.63–4.18, *p* < 0.001) and 3.03 (95%, 1.70–4.36, *p* < 0.001), as presented in Table 2 and Figure 2A.

**Table 2:** Results of the main outcome indicators.

(ODI) Time	Mean (SD)		Mean difference from baseline (95% CI), *p* value		Differences between groups		Group × time interaction	Time	Group
	TCE group (n = 83)	Tuina + TCE group (n = 83)	TCE group	Tuina + TCE group	Mean difference (95% CI)	*p* value			
Baseline	24.02 (3.68)	24.06 (3.27)	NA	NA	−0.04 (−1.10, 1.03)	0.948	*F* = 27.047, *p* < 0.001, partial *η*^2^ = 0.332	*F* = 268.313, *p* < 0.001, partial *η*^2^ = 0.832	*F* = 23.957, *p* < 0.001, partial *η*^2^ = 0.127
4 weeks	20.23 (3.43)	16.31 (4.18)	3.79 (2.92, 4.67), <0.001	7.75 (6.88, 8.62), <0.001	3.92 (2.75, 5.09)	<0.001			
12 weeks	16.92 (3.94)	14.01 (4.38)	7.11 (5.89, 8.33), <0.001	10.05 (8.83, 11.26), <0.001	2.90 (1.63, 4.18)	<0.001			
24 weeks	17.29 (3.98)	14.26 (4.69)	6.74 (5.36, 8.11), <0.001	9.80 (8.43, 11.16), <0.001	3.03 (1.70, 4.36)	<0.001			

SD, Standard deviation; NA, Not applicable; ODI, Oswestry Disability Index.

Secondary outcome indicators were statistically analysed. After 4 weeks of intervention, the Tuina + TCE group had significantly better outcomes than the TCE group. In the SF-MPQ scale, all scores in the Tuina + TCE group were lower than those in the TCE group, with mean differences of VAS, 0.86 (95%, 0.53–1.19, *p* < 0.001); PRI, 1.09 (95%, 0.41–1.78, *p* = 0.002); PPI, 0.45 (95%, 0.24–0.66, *p* < 0.001). In the SF-36 scale, all scores were higher in the Tuina + TCE group than in the TCE group, with mean differences of PF, −4.92 (95%, −6.96−−2.88, *p* < 0.001); MH, −3.77 (95%, −6.38−−1.16, *p* = 0.005). At weeks 12 and 24, the differences in mean MH scores between the Tuina + TCE group and the TCE group were −2.59 (95%, −5.09−−0.10, *p* = 0.042) and −2.39 (95%, −5.03–0.24, *p* = 0.075), which were not statistically significant. For the remaining outcomes, the superiority of the Tuina + TCE group persisted at 12- and 24-week follow-ups (Table 3; Figures 2B–F).

**Table 3:** Results for secondary outcome indicators.

Time	Mean (SD)		Mean difference from baseline (95% CI), *p* value		Differences between groups		Group × time interaction	Time	Group
	TCE group (n = 83)	Tuina + TCE group (n = 83)	TCE group	Tuina + TCE group	Mean difference (95% CI)	*p* value			
VAS									
Baseline	5.08 (1.03)	5.02 (1.06)	NA	NA	0.06(−0.26, 0.38)	0.698	*F* = 11.432, *p* < 0.001, partial *η*^2^ = 0.065	*F* = 569.968, *p* < 0.001, partial *η*^2^ = 0.776	*F* = 17.722, *p* < 0.001, partial *η*^2^ = 0.097
4 weeks	3.94 (1.13)	3.08 (1.01)	1.15 (0.96, 1.33), <0.001	1.94 (1.76, 2.12), <0.001	0.86(0.53, 1.19)	<0.001			
12 weeks	2.73 (1.13)	2.10 (0.95)	2.35 (2.04, 2.65), <0.001	2.92 (2.62, 3.23) < 0.001	0.64(0.32, 0.96)	<0.001			
24 weeks	2.81 (1.08)	2.08 (1.03)	2.27 (1.92, 2.63), <0.001	2.94 (2.58, 3.29), <0.001	0.72(0.40, 1.05)	<0.001			
SF-MPQ (PRI)									
Baseline	12.34 (2.21)	12.46 (2.18)	NA	NA	−0.13 (−0.80, 0.54)	0.709	*F* = 6.260, *p =* 0.001, partial *η*^2^ = 0.037	*F* = 336.558, *p* < 0.001, partial *η*^2^ = 0.671	*F* = 4.501, *p* = 0.035, partial *η*^2^ = 0.027
4 weeks	9.80 (2.27)	8.70 (2.23)	2.54 (2.09, 2.99), <0.001	3.76 (3.31, 4.21), <0.001	1.09 (0.41, 1.78)	0.002			
12 weeks	8.55 (2.33)	7.73 (2.53)	3.78 (3.17, 4.40), <0.001	4.74 (4.12, 5.35), <0.001	0.83 (0.09, 1.57)	0.029			
24 weeks	8.64 (2.48)	7.73 (2.87)	3.70 (2.97, 4.42), <0.001	4.74 (4.02, 5.46), <0.001	0.91 (0.09, 1.73)	0.029			
SF-MPQ (PPI)									
Baseline	2.75 (0.71)	2.69 (0.58)	NA	NA	0.06 (−0.14, 0.26)	0.575	*F* = 11.418, *p* < 0.001, partial *η*^2^ = 0.065	*F* = 188.052, *p* < 0.001, partial *η*^2^ = 0.553	*F* = 16.632, *p* < 0.001, partial *η*^2^ = 0.092
4 weeks	2.06 (0.79)	1.61 (0.56)	0.69 (0.55, 0.83), <0.001	1.08 (0.94, 1.22), <0.001	0.45 (0.24, 0.66)	<0.001			
12 weeks	2.05 (0.75)	1.60 (0.62)	0.70 (0.52, 0.88), <0.001	1.09 (0.92, 1.27), <0.001	0.45 (0.24, 0.66)	<0.001			
24 weeks	2.18 (0.72)	1.65 (0.74)	0.57 (0.37, 0.76), <0.001	1.04 (0.84, 1.23), <0.001	0.53 (0.30, 0.75)	<0.001			
SF-36 (PF)									
Baseline	49.82 (6.69)	49.76 (6.81)	NA	NA	0.06 (−2.00, 2.12)	0.956	*F* = 12.195, *p* < 0.001, partial *η*^2^ = 0.069	*F* = 205.642, *p* < 0.001, partial *η*^2^ = 0.555	*F* = 16.315, *p* < 0.001, partial *η*^2^ = 0.090
4 weeks	56.69 (6.59)	61.61 (6.77)	−6.87 (−8.23, −5.50), <0.001	−11.85 (−13.20, −10.49), <0.001	−4.92 (−6.96, −2.88)	<0.001			
12 weeks	59.70 (7.71)	64.82 (7.74)	−9.88 (−11.92, −7.84), <0.001	−15.06 (−17.09, −13.03), <0.001	−5.12 (−7.48, −2.76)	<0.001			
24 weeks	58.40 (10.47)	64.62 (9.36)	−8.58 (−11.52, −5.63), <0.001	−14.86 (−17.78, −11.93), <0.001	−6.22 (−9.26, −3.19)	<0.001			
SF-36 (MH)									
Baseline	54.60 (8.89)	53.98 (8.09)	NA	NA	0.63 (−1.97, 3.22)	0.635	*F* = 8.799, *p* < 0.001, partial *η*^2^ = 0.051	*F* = 266.941, *p* < 0.001, partial *η*^2^ = 0.618	*F* = 2.915, *p* = 0.090, partial *η*^2^ = 0.017
4 weeks	61.59 (9.12)	65.36 (7.93)	−6.99 (−8.31, −5.66), <0.001	−11.38 (−12.70, −10.06), <0.001	−3.77 (−6.38, −1.16)	0.005			
12 weeks	64.19 (8.77)	66.79 (7.52)	−9.59 (−11.64, −7.54), <0.001	−12.81 (−14.85, −10.77), <0.001	−2.59 (−5.09, −0.10)	0.042			
24 weeks	63.25 (9.48)	65.64 (7.68)	−8.65 (−10.84, −6.46), <0.001	−11.67 (−13.84, −9.49), <0.001	−2.39 (−5.03, 0.24)	0.075			

SD, Standard deviation; NA, Not applicable; VAS, Visual Analogue Scale; SF-36: Short Form of Quality of Life; SF-MPQ: Short-Form McGill Pain Questionnaire; PRI, Pain rating index; PPI: Present pain intensity; PF, Physical function; MH, Mental health.

### 3.2 Gait analysis

76 subjects from the main centre participated in the gait analysis. At baseline, no significant differences were noted in gait parameters between the two groups (*p* > 0.05). After 4 weeks of intervention, all gait parameters changed in the Tuina + TCE group and the TCE group, with no statistically significant difference between the two groups in terms of changes in step length and stride length. Significant changes in cadence and double-stance phase occurred in the Tuina + TCE group compared with the TCE group, with a mean difference in cadence of −3.95 (95%, −7.81−−0.09, *p* = 0.045) and a mean difference in double-stance phase of 0.42 (95%, 0.02–0.80, *p* = 0.039), as presented in Table 4.

**Table 4:** Comparison of gait parameters between the two groups.

Time	Mean (SD)		Mean difference from baseline (95% CI)		Differences between groups	
	TCE group (n = 38)	Tuina + TCE group (n = 38)	TCE group	Tuina + TCE group	Mean difference (95% CI)	*p* value
Step length (m)						
Baseline	0.62 (0.06)	0.61 (0.07)	NA	NA	0.01 (−0.03, 0.04)	0.689
4 weeks	0.63 (0.06)	0.63 (0.08)	−0.01 (−0.01, −0.01)	−0.02 (−0.02, −0.01)	−0.01 (−0.04, −0.03)	0.767
Stride length (m)						
Baseline	1.24 (0.12)	1.22 (0.15)	NA	NA	0.02 (−0.04, 0.08)	0.053
4 weeks	1.25 (0.12)	1.26 (0.15)	−0.02 (−0.03, −0.01)	−0.05 (−0.06, −0.03)	−0.01 (−0.07, 0.05)	0.742
Cadence (step/min)						
Baseline	102.11 (9.45)	102.32 (8.12)	NA	NA	−0.21 (−4.24, 3.81)	0.917
4 weeks	104.84 (8.98)	108.79 (7.86)	−2.74 (−3.54, −1.93)	−6.47 (−7.52, −5.43)	−3.95 (−7.81, −0.09)	0.045
Double-stance phase (%)						
Baseline	23.05 (1.61)	23.18 (1.25)	NA	NA	−0.13 (−0.68, 0.42)	0.636
4 weeks	22.29 (0.98)	21.87 (0.74)	0.76 (0.57, 0.96)	1.32 (1.04, 1.59)	0.42 (0.02, 0.80)	0.039

SD, Standard deviation; NA, Not applicable.

### 3.3 Safety outcomes

Throughout the study period, no serious adverse events were reported in either group. A small number of participants in the Tuina combined with TCE group reported mild, transient discomfort (e.g., local muscle soreness) following Tuina sessions, which resolved spontaneously without intervention. No participants discontinued treatment due to adverse events. These findings suggest that both Tuina and TCE interventions were safe and well tolerated.

## 4 Discussion

The present study evaluated the effects of active exercise combined with passive manipulative therapy on functional disability, pain, quality of life, and gait in patients with LDH by comparing the clinical efficacy of Tuina in combination with TCEs versus TCEs alone. The results demonstrated that the combined intervention led to significantly greater improvements in functional disability and gait performance, particularly in cadence and double-stance phase. Beyond pain relief and quality-of-life enhancement, the combination therapy conferred additional advantages, which were maintained during long-term follow-up.

Tuina, a complementary therapy for LDH, excels in pain relief and functional restoration (30). For instance, a recent RCT demonstrated that massage reduced symptoms in LDH patients with radiculopathy, with sustained improvements at 9 months (31). Furthermore, an functional MRI study revealed that spinal manipulative therapy modulates brain function in LDH patients, highlighting potential central mechanisms underlying its clinical efficacy (32). Another study compared Tuina therapy with lumbar traction and found that patients with LDH in the Tuina group experienced marked improvements in pain and disability, reduced muscle tension in the lower back, and decreased serum levels of inflammatory factors. These findings suggest that Tuina not only alleviates pain and restores function but also relieves muscle tension and reduces inflammatory responses (33). However, based on our clinical observations, although Tuina therapy effectively relieved symptoms in patients with LDH, it showed no significant benefit in preventing recurrence of LDH, nor did it enhance the strength of the lumbar muscles or improve spinal stability. TCE, such as Taiji, Baduanjin, and Wuqinxi, have a long history in China. They are used for both prevention and treatment of a wide range of conditions, including diabetes mellitus, coronary heart disease, hypertension, and LDH, and are known to benefit both physical and mental health. TCEs also exert certain effects on pain relief and functional recovery (34). A systematic review demonstrated that TCEs are effective in alleviating musculoskeletal pain, particularly in improving back and knee dysfunction and stiffness (35). In addition to pain relief, TCEs can enhance lumbar stability, improve muscle strength, and promote balance control and flexibility (36–38). These advantages allow TCEs to effectively compensate for the limitations of Tuina therapy, making the combination of Tuina and TCEs a more favorable approach for the management of LDH.

Consistent with previous studies (21, 39–40), our findings support the superiority of combination therapy over monotherapy, despite potential biases inherent in the intervention modality. Our study further contributes to this body of knowledge by providing multicentre evidence that combining these two modalities results in significantly greater improvements in function, pain, and gait, with effects sustained over 24 weeks. Different from these studies, in addition to observing the short-term efficacy, up to 24 weeks of follow-ups were performed to assess the long-term efficacy of nudging combined with TCEs. In addition, the effect of treatment with nudging combined with TCEs on dysfunction in patients with LDH was further assessed by gait analysis. The results of the gait analysis show that the two groups of patients had greater changes only in cadence and double-stance phase after about 4 weeks, with smaller differences in step length and stride length. This outcome may be related to the fact that only gait values for 76 subjects at the main centre were measured, and they were not evaluated during follow-up. Nonetheless, the group of Tuina combined with TCEs also significantly increased the patients’ step frequency, shortened the double-support phase time and accelerated the patients’ step speed after 4 weeks of treatment. In addition, no significant difference was observed in the MH scores between the Tuina combined with TCE group and the TCE group at the 24th week of follow-up. This result suggests that the long-term efficacy of the combination treatment is not superior to single TCE treatment in improving mental health. Nevertheless, Tuina combined with TCEs remains more favourable in terms of long-term efficacy.

The synergistic mechanism of action may be attributable to their complementary physiological effects. Tuina likely exerts its benefits through biomechanical stimulation of soft tissue, modulation of the pain-gating mechanism, enhancement of local blood flow, and downregulation of inflammatory cytokines (41–43). Meanwhile, TCEs—through slow, controlled movements coordinated with breathing and posture—may improve core muscle endurance, balance control, proprioception, and mind–body integration (44). Together, these interventions may promote both structural and functional rehabilitation of the lumbar spine, resulting in more comprehensive recovery than either modality alone.

This study has several limitations that should be acknowledged. First, due to the nature of the intervention, blinding of participants and therapists was not feasible, which may have introduced performance bias; only outcome assessors were blinded. Although centralized training and standardized treatment manuals were provided, and evaluator blinding was implemented, some inter-centre and therapist-related heterogeneity in intervention delivery may still exist. Second, although participants were instructed to refrain from additional exercise, individual lifestyle habits—such as household chores, walking, or cycling—may have led to differences in physical activity levels across participants. Third, each participant’s learning ability and adherence to TCE practice varied, making it difficult to ensure consistent treatment intensity. Additionally, the sample was drawn exclusively from four urban Chinese hospitals, potentially limiting the generalizability of the findings to other populations or rural areas. Finally, although clinical outcomes such as ODI, SF-36, and gait analysis were measured, no biological or imaging indicators were included to objectively assess the mechanistic effects of the interventions.

Despite these limitations, the use of standardized intervention protocols and an extended follow-up period enhances the internal validity of our findings. The treatment was well tolerated, with no serious adverse events reported and only a few participants experiencing mild, transient discomfort following Tuina therapy. This favorable safety profile underscores the feasibility and clinical acceptability of incorporating Tuina and TCEs into routine care for patients with LDH. Future research should aim to validate these findings in more diverse populations, incorporate objective physiological or biomarker-based assessments to clarify underlying mechanisms, and evaluate cost-effectiveness as well as the long-term sustainability of therapeutic benefits. Such efforts would support the broader integration of combined Tuina and TCE therapy into clinical practice worldwide.

## 5 Conclusion

The combination of Tuina and TCEs in the treatment of LDH improved patients’ pain and disability more than TCEs alone, and provided better improvements in patients’ quality of life and gait, and this advantage persisted at week 24. The combination of Tuina and TCEs should be considered in the treatment of symptomatic LDH.

## Data Availability

The raw data supporting the conclusions of this article will be made available by the authors, without undue reservation.
